# Anti-Electrostatic Anion-Anion Noncovalent Interactions Are Not Halogen Bonds: Evidence from X···O Contacts in XO_4_^−^ Dimers and Oligomers in Crystals Structures

**DOI:** 10.3390/ijms27125267

**Published:** 2026-06-10

**Authors:** Arpita Varadwaj, Pradeep R. Varadwaj, Helder M. Marques, Bogumiła Jezierska, Ireneusz Grabowski, Mohd. Mudassir Husain, Koichi Yamashita

**Affiliations:** 1 Department of Chemical System Engineering, School of Engineering, The University of Tokyo, 7-3-1, Tokyo 113-8656, Japan; 2 Institute of Physics, Faculty of Physics, Astronomy, and Informatics, Nicolaus Copernicus University in Toruń, 87-100 Toruń, Poland; 3 Molecular Sciences Institute, School of Chemistry, University of the Witwatersrand, Johannesburg 2050, South Africa; 4 Institute of Advanced Studies, Nicolaus Copernicus University in Toruń, ul. Wileńska 4, 87-100 Toruń, Poland; 5 Department of Applied Sciences and Humanities, Faculty of Engineering and Technology, Jamia Millia Islamia, New Delhi 110025, India

**Keywords:** anion-anion assemblies, anti-electrostatic noncovalent interactions, physical chemistry and chemical physics, geometries, energies, MESP-QTAIM-NBO-IGMH analyses, environmental effects

## Abstract

This study investigates anion–anion assemblies involving perhalate anions, XO_4_^−^ (X = Cl, Br, I), in crystal structures retrieved from the Cambridge Structural Database to clarify the nature of the intermolecular interactions frequently interpreted as halogen bonds. Molecular electrostatic surface potential analysis demonstrates that isolated XO_4_^−^ anions do not exhibit electrophilic σ-holes on the halogen or oxygen atoms along the O–X bond extensions, thereby precluding their role as conventional halogen- or chalcogen-bond donors. Gas-phase calculations further show that direct anion–anion assemblies are intrinsically repulsive and unstable in isolation. However, when dielectric screening is introduced through implicit solvation models, metastable dimeric and oligomeric arrangements consistent with crystallographic motifs become accessible. Complementary QTAIM, IGMH, NBO, and SAPT analyses show that the observed X···O and O···O contacts are weak, environment-assisted anti-electrostatic interactions arising from a combination of dielectric screening, polarization, dispersion, and donor–acceptor contributions. The results demonstrate that the structural organization of perhalate anions in crystalline environments is governed primarily by collective environmental and crystal-packing effects rather than intrinsic attractive interactions between isolated anions.

## 1. Introduction

Noncovalent interactions underpin molecular recognition and supramolecular assembly, yet not all such contacts conform to simple electrostatic expectations [1,2], and some may fall within the domain of coordinate bonding [3]. A particularly intriguing case is the association of like-charged species [4,5], where classical coulombic arguments predict strong repulsion but experiments and computations nonetheless reveal stable or metastable aggregates [6,7,8,9]. In this context, anion–anion contacts—especially between halogen oxyanions such as XO_3_^−^ and XO_4_^−^ (X = Cl, Br, I)—have attracted attention because crystallographic data frequently show directional X···O contacts, leading to dimers, chains, and higher-dimensional architectures [4,10,11]. These observations appear to contradict intuitive electrostatics and suggest that additional effects such as polarization, dispersion, and environmental stabilization play a crucial role in enabling anti-electrostatic assembly [5,12,13].

Halogen bonding, regardless of whether the halogen derivative is hypervalent or not, has been used to rationalize directional halogen-centered contacts [2,14] typically defined as attractive interactions between an electron-poor region (commonly a σ-hole) on a halogen atom and an electron-rich site on a neighboring species [15,16]. A σ-hole on the electrostatic surface of an atom A is an electron-density-deficient region lying opposite to, along the outermost extension of an R–A covalent bond, where R is the remainder of the molecular entity [17,18,19]. The electrostatic potential that quantifies it can be negative or positive [20,21,22].

While the σ-hole framework successfully explains intermolecular interactions and the resulting ordering of monomeric entities in many neutral systems, [2,16,17] it becomes less straightforward when both interacting partners are anions, despite the formation of ordered chains, ribbons, layers, or cage-like structures in the crystalline phase [4,10,11]. In such cases, halogen-centered contacts may still appear highly directional, yet they lack a clear electrophilic site and may instead involve anisotropic electron density, lateral interactions, or regions not associated with a classical σ-hole. This raises a fundamental question: when long-ranged, directional X···O contacts occur between two negatively charged species, should they be classified as conventional halogen bonds [4,10,11], or more appropriately as manifestations of halogen-centered anti-electrostatic interactions stabilized by their environment?

Here we investigate representative XO_4_^−^ dimers and oligomeric motifs using density functional theory at the M06-2X level of theory [23,24] combined with symmetry-adapted perturbation theory [25,26] to clarify the physical origin of these anion–anion contacts. We investigate the nature of anion–anion X···O contacts in halogen oxyanion systems, with emphasis on the role of environment in their stabilization. We examine whether intrinsic gas-phase interactions between anions are feasible, given the strong electrostatic repulsion that would be expected to drive such systems toward dissociation. We further analyze energetic components based on solution-phase geometries to assess how solvent effects, polarization, and dispersion contributions influence the observed interaction patterns. This approach allows us to assess the extent to which reported anion–anion X···O contacts in halogen oxyanion solids reflect intrinsic halogen bonding or instead represent environment-assisted, halogen-centered anti-electrostatic associations governed by a balance of weak noncovalent forces. We employ molecular electrostatic surface potential (MESP) [1,27,28], NBO’s second-order charge-transfer [29], quantum theory of atoms in molecules (QTAIM) [30], and independent gradient model based on Hirshfeld partitioning (IGMH) [31] analyses to evaluate these interactions.

Before presenting the results for the XO_4_^−^ dimers and oligomeric systems, it is necessary to clarify the conceptual framework underlying the classification of noncovalent interactions involving halogen-containing anions. Since the present study critically examines whether the observed anion–anion contacts should be interpreted as halogen bonds or as a different class of interaction, Section 2 briefly summarizes the current definitions of halogen bonding and related noncovalent interactions, together with the role of σ-hole characteristics and their electrostatic nature. This background is essential for establishing the terminology and theoretical context used throughout the subsequent discussion of the results.

## 2. Definition of the Halogen Bond, and Other Noncovalent Interactions

We have recently proposed a definition of the halogen bond [32]:

*A halogen bond (HaB) develops in a chemical system when a net attractive engagement occurs between an electron-density-deficient (electrophilic) region on the electrostatic surface of a halogen atom in a molecular entity and a close-lying electron-density-rich (nucleophilic) region on the electrostatic surface of the same or another identical or different molecular entity.*

The definition is consistent with the IUPAC definition of a halogen bond that was put forward in 2013, a definition that was proposed just a year after the revised definition of the hydrogen bond. Their wording is very similar.

*A halogen bond occurs when there is evidence of a net attractive interaction between an electrophilic region associated with a halogen atom in a molecular entity and a nucleophilic region in another, or the same, molecular entity* [33]*.*

*The hydrogen bond is an attractive interaction between a hydrogen atom from a molecule or a molecular fragment X–H, in which X is more electronegative than H, and an atom or a group of atoms in the same or a different molecule, in which there is evidence of bond formation* [34]*.*

Clearly, the formation of an intermolecular bond involves a donor that is electrophilic, and the name of the element or group is used in naming the bond [32,35]. These definitions primarily address σ-hole donors located on covalently bound hydrogen or halogen atoms in molecular species that interact with nucleophiles. In this context, the σ-hole is electrophilic rather than nucleophilic. However, the definitions may also be extended to *p*-hole and π-hole interactions, provided that the halogen derivative is capable of exhibiting such features and that they are likewise electrophilic.

Arguments have been advanced to classify intermolecular interactions between anions (e.g., ICl_4_^−^) as halogen bonds [36]. Others have noted that a halogen bond does not necessarily require a positive site to interact with a negative site [37]. Some studies [4] have also modified the fundamental definition to recognize interactions between negative σ-holes on a molecular anion and positive oxygen sites in an entirely cationic species as halogen bonds. It is therefore questionable whether such interactions should truly be classified as halogen bonds within the framework of a dimer approximation between the interacting monomers, or whether they are better described as an unusual class of chalcogen bond. Our current investigation, which will be discussed in a forthcoming article, however, demonstrates that the interaction is a chalcogen bond, as defined by the IUPAC working group [38]:

*A chalcogen bond (ChB) is a net attractive interaction between an electrophilic region associated with a chalcogen atom in a molecular entity and a nucleophilic region in another, or the same, molecular entity.*

## 3. Results

### 3.1. Negative σ-Hole and Its Directional Bonding Capability

**Figure 1:**
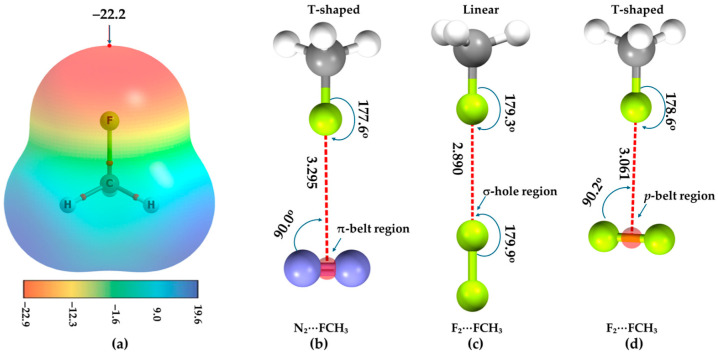
(**a**) QTAIM molecular graph of CH_3_F superimposed on its MESP map. The binary complexes shown are (**b**) N_2_⋯FCH_3_, (c) F_2_⋯FCH_3_, and (**d**) F_2_⋯FCH_3_. In (**a**), the electrostatic potential values (kcal mol^−1^) are indicated, and the maximum positive potential on the F atom is marked by a small red circle. Selected bond lengths and angles in (**b**–**d**) are given in Å and degrees, respectively. The bonding regions on N_2_ and F_2_ in (**b**) and (**d**) are indicated by small red spheres, respectively, and the dotted lines represent noncovalent interactions.

The electrostatic surface of fluorine along the outer extension of the C–F bond in H_3_C–F is negative (*V_S_*_,max_ = −22.2 kcal mol^−1^) in the gas phase as shown in Figure 1a; nevertheless, it exhibits directional bonding propensity when placed near positive regions. In all three complexes shown in Figure 1b–d, the intermolecular interactions are directed along the extension of the negative σ-hole on the C–F bond, though the precise directionality is determined by the electrophilic character of the bond donor atom or bonding region, such as the electrophilic π-density associated with the N_2_ triple bond. We therefore characterize the (N_2_)_mid-point_⋯F interaction in the T-shaped complex N_2_⋯FCH_3_, Figure 1b, as a π-centered pnictogen bond rather than an F-centered σ-hole-driven halogen bond.

A similar directional behavior is observed when two σ-holes on different molecular entities interact to form the linear complex shown in Figure 1c, with one σ-hole negative and the other positive, resulting in an attractive intermolecular interaction. This corresponds to a σ-hole-driven halogen bond involving the electrophilic terminal region of the fluorine atom in F_2_, as exemplified by the linearly bonded F_2_⋯FCH_3_ complex.

The geometric stability of the T-shaped F_2_⋯FCH_3_ dimer in Figure 1d is consistent with the formation of an (F_2_)_mid-point_⋯F halogen bond, where the *p*-type electrophilic belt of F_2_ donates a halogen bond to the negative σ-hole of F in H_3_C–F. This interaction should not be interpreted as a σ-hole-centered halogen bond.

Symmetry-adapted perturbation theory (SAPT) analysis [25,26] was performed at the SAPT2+3(CCD) level for all complexes. Energy decomposition analysis was performed on the energy-minimized geometries, partitioning the total interaction energy into electrostatic, exchange, induction, and dispersion contributions. Across all systems, dispersion constitutes the dominant stabilizing component, with induction providing a smaller additional contribution (cf. Table 1). The T-shaped F_2_⋯FCH_3_ complex is the most stable (−0.64 kcal mol^−1^), reflecting a favorable interplay between dispersion and well-aligned electrostatics, whereas the linear F_2_⋯FCH_3_ arrangement is the least stable (−0.22 kcal mol^−1^), demonstrating that geometry critically governs the balance between electrostatic and dispersive interactions.

Second-order NBO analysis reveals the following hyperconjugative interactions for the linear complex in Figure 1c: σ(C–F) → RY*(3) F(F_2_), LP(1) F → RY* F(F_2_), as well as σ(F–F) → RY*(3) F(CH_3_F) and σ(F–F) → RY*(4) F(CH_3_F), where σ, LP, and RY* denote the σ-bonding, lone-pair bonding, and Rydberg-type antibonding orbitals, respectively. The corresponding stabilization energies *E*^(2)^ are 0.38, 0.12, 0.12, and 0.21 kcal mol^−1^, respectively. For the T-shaped dimer in Figure 1d, among several other weak donor–acceptor interactions with *E*^(2)^ values between 0.05 and 0.10 kcal mol^−1^, the strongest hyperconjugative charge-transfer interaction involves σ(C–F) → RY*(1) F(F_2_), with an *E*^(2)^ of 0.73 kcal mol^−1^, which may be described as a *p*-belt interaction.

In the case of the N_2_⋯FCH_3_ interaction (Figure 1b), the charge-transfer delocalization involves donor–acceptor interactions such as LP(2) or LP(3) on F → RY*(2)/RY*(3) N on the nitrogen atoms of N_2_, as well as π(N_2_) → RY*(3) on F. The corresponding *E*^(2)^ are small and lie in the range of approximately 0.07–0.08 kcal mol^−1^ and 0.06 kcal mol^−1^, respectively, indicating weak but measurable donor–acceptor mixing characteristic of the noncovalent interaction.

### 3.2. Monomer Anions and Their Molecular Electrostatic Potential

**Figure 2:**
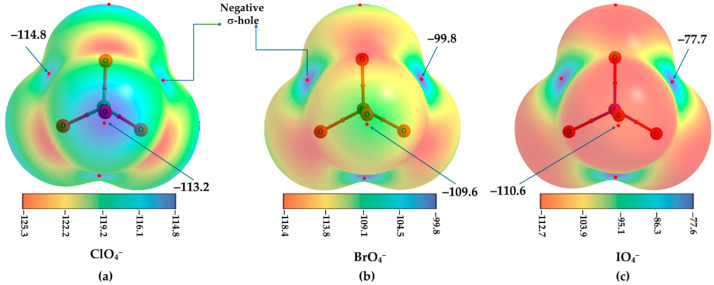
M06-2X/SMD-level QTAIM topology of bonding superimposed on the MESP plot for (**a**) ClO_4_^−^, (**b**) BrO_4_^−^, and (**c**) IO_4_^−^ anions. Selected maxima of the electrostatic potential (kcal mol^−1^) are indicated by arrows, with small circles marking the potential maxima on I and X. The 0.001 a.u. isodensity envelope was used to map the potential.

The XO_4_^−^ (X = Cl, Br, I) anions exhibit σ-holes both on the halogen derivative and oxygen atoms: four on X along the extensions of the O–X bonds, and four on O along the extensions of the X–O bonds. For example, in SMD water, the *V_S_*_,max_ values for these σ-holes are −114.8, −99.8, and −77.6 kcal mol^−1^ on Cl, Br, and I, respectively, whereas the corresponding values on O are −113.2, −109.6, and −110.6 kcal mol^−1^ for ClO_4_^−^, BrO_4_^−^, and IO_4_^−^, respectively. In the gas phase, the *V_S_*_,max_ values on the halogen centers are −114.1, −100.5, and −83.4 kcal mol^−1^ for the corresponding species, while those on O are −112.6, −108.9, and −108.4 kcal mol^−1^, respectively. Figure 2a–c shows the MESP plots of XO_4_^−^.

A discrepancy is observed in the trend of the electrostatic potential maxima (σ-hole strength) on O along the X–O bond extensions across the halogen series: in the gas phase the trend is I < Br < Cl, whereas in the SMD (solution) model it is Br < I < Cl. The reversal of the Br and I maxima in solution likely arises from differential stabilization of oxygen lone pairs by the polarizable continuum, which affects larger halogens more strongly than Cl. Previous reports on this system at the PBE0-D3/TZVP level show symmetric σ-holes on I with a *V_S,_*_max_ value of −87.8 kcal mol^−1^ [4]. The differences between the present results and earlier findings arise not only from the different exchange–correlation treatments employed but also from the size and quality of the basis sets. The current study uses the larger def2-TZVPPD basis set, which is particularly important for accurately describing heavy atoms such as iodine.

### 3.3. Dimeric and Oligomeric Anions

#### 3.3.1. The Dimers and Oligomers of ClO_4_^−^

**Figure 3:**
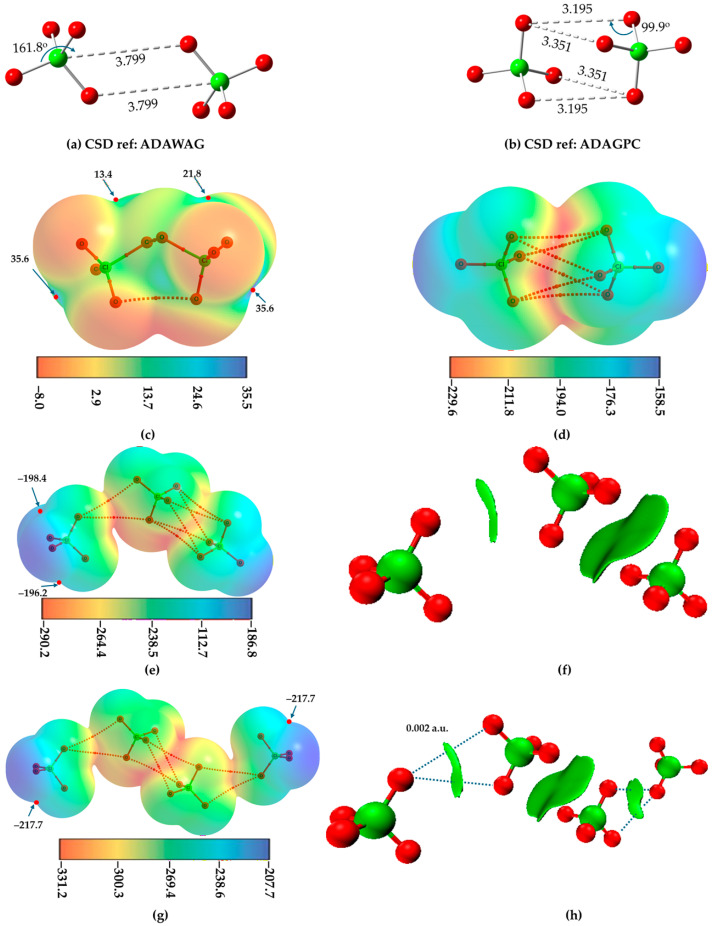
Geometry of the (ClO_4_^−^)_2_ dimer extracted from the crystals of (**a**) [C_30_H_32_Cd_2_Cl_2_N_8_]^2+^·2(ClO_4_^−^) and (**b**) [C_10_H_12_Ag_2_N_10_]^4+^·4(ClO_4_^−^)·2(H_2_O), showing the intermolecular separation and spatial arrangement of the anions. (**c**,**d**) present the QTAIM topologies superimposed on the MESP plots obtained on the energy-minimized geometries of the corresponding dimers in SMD. (**e**,**g**) The corresponding graphs for (ClO_4_^−^)_3_ and (ClO_4_^−^)_4_, respectively, whereas (**f**,**h**) show the IGMH plots of the same systems. Bond paths and bond critical points in (**c**,**d**) and (**e**,**g**) are shown as solid and dotted lines in atom colors, respectively, with potential values given in kcal mol^−1^. Selected bond distances and angles in (**a**,**b**) are in Å and degrees, respectively, while the maxima of the electrostatic potential are indicated as small red circles in (**c**,**e**,**g**), marked by arrows. The dotted lines in black in (**h**) are manually constructed to indicate attractive interactions.

Two geometries of (ClO_4_^−^)_2_ extracted from crystal structures with CSD codes ADAWAG and ADAGPC were fully optimized in solution using the SMD model, yielding the structures shown in Figure 3a,b. In the former, reorganization between the monomers leads to formation of an O–O covalently bridged Cl_2_O_8_^2−^ dianion (*r*(O–O) = 1.385 Å), whereas in the latter the two ClO_4_^−^ units remain non-covalently associated. These structures are not conformers, as evidenced by a large energy difference of 332 kcal mol^−1^, with the non-covalently bound arrangement lower in energy. The latter is therefore energetically preferred in solution, while formation of the coordinated Cl_2_O_8_^2−^ dianion is significantly less favorable. This arises because covalent coupling to form Cl_2_O_8_^2−^ distorts the symmetry of the highly stabilized tetrahedral ClO_4_^−^ units, reduces charge delocalization, and concentrates negative charge within a single dianionic framework, resulting in increased intramolecular coulomb repulsion and less effective solvation of the localized charge.

QTAIM molecular graphs (Figure 3c,d) reveal O···O close contacts in both Cl_2_O_8_^2−^ and (ClO_4_^−^)_2_ that are not chalcogen bonds, with one contact identified in the former and six in the latter based on the bond-path and bond-critical-point topologies (see the dotted lines). In Cl_2_O_8_^2−^, the contact is intramolecular (*r*(O···O) = 2.834 Å), whereas in (ClO_4_^−^)_2_ the contacts are intermolecular (*r*(O···O) values 3.485, 3.512, and 3.514 Å). These longer distances indicate elongation due to increased electrostatic repulsion relative to the crystal values of 3.195 and 3.351 Å (Figure 3b), yet they still correspond to genuine intermolecular close contacts, as evidenced by the overlap of the MESP surfaces. This is also supported by SAPT results obtained on the solvated dimer geometry in the gas phase: the electrostatic component is 73.8 kcal mol^−1^, exceeding exchange (2.1 kcal mol^−1^), induction (−4.0 kcal mol^−1^), and dispersion (−3.0 kcal mol^−1^), giving a total interaction energy of 69.0 kcal mol^−1^.

The (ClO_4_^−^)_2_ dimer features two nearly equivalent Cl···O close contacts, with *r*(Cl···O) values of 3.934 and 3.944 Å and O–Cl···O angles of 159.8 and 159.9°, suggesting that the intermolecular interaction is (negative) σ-hole-centered, but does not represent a halogen bond.

MESP analysis shows that each chlorine atom in Cl_2_O_8_^2−^ hosts four electrophilic σ-holes with *V_S_*_,max_ values of 35.6, 13.4, 21.8, and 22.9 kcal mol^−1^. The bridging O–O region is also entirely electrophilic, both along bond extensions and around the O atoms, with *V_S_*_,max_ values of 9.8 and 17.4 kcal mol^−1^, enabling participation in halogen and chalcogen bonding, respectively.

The (ClO_4_^−^)_2_ dimer in Figure 3d features four σ-holes on the O atoms of each anion; three are off-axis and one lies along the Cl–O bond extension (*V_S,max_* = −158.6 and −178.2 to −178.6 kcal mol^−1^, respectively). The latter three are off-axis opposite to the noncovalent bonding direction. No σ-hole is observed on the surface of Cl atoms at the 0.001 a.u. isodensity envelope. This absence arises from the arbitrary nature of this envelope, which approximately represents the van der Waals surface for many molecular systems and, in this case, lies relatively far from the Cl nucleus. Consequently, any σ-hole on Cl may very well not be captured at this density.

Following the recommendation of Bader and co-workers [39] that suggest the use of 0.001 and 0.002 a.u. isodensity envelopes that cover 95% of the molecular properties, we therefore examined slightly higher isodensity envelopes (0.0013 and 0.0015 a.u.). At these values, σ-holes become apparent on the Cl atoms in the ClO_4_^−^ monomers in the dimer configuration, confirming that they are genuine features of the molecular electrostatic potential rather than artifacts of the chosen isodensity surface. For instance, each monomer in the dimer features three σ-holes when the 0.0013 a.u. isodensity envelope was used to map the potential, while the fourth is effectively annihilated in the intermolecular bonding region. The σ-hole strengths are −176.3, −176.4, and −176.5 kcal mol^−1^ on each monomer, indicating that the electrostatic surface of the monomer becomes more negative in the dimer relative to its isolated state (see Figure 2a). Consequently, the dimer cannot serve as an electrophilic halogen-bond donor toward another identical anion in close proximity and therefore cannot form a halogen bond.

Figure 3e,f and Figure 3g,h shows the (ClO_4_^−^)_3_ and (ClO_4_^−^)_4_ oligomers, respectively. The overall skeletal framework and directional orientations of the anions are largely preserved compared to the crystal structures; however, the intermolecular distances are appreciably longer than in the crystal structure. In the latter (CSD ref: ADAGPC), the O···O close contacts between monomers show significant variability: for one pair of monomers, the distances are 3.030 and 3.178 Å, while for another pair, they are 3.290 and 3.330 Å. In the SMD-optimized geometry of (ClO_4_^−^)_4_, these distances increase to 3.771 [3.766] and 3.800 [3.934] Å for the first pair, and 3.593 [3.661], [3.539, 3.534, 3.550, 3.560] 3.506, and [3.680] 3.714 Å for the second pair, where the values in the square brackets are for (ClO_4_^−^)_3_. This indicates that both in the solid state and in solution, the intermolecular separations between the central monomers are shorter than those between the terminal pairs. This elongation likely arises from increased electrostatic and exchange repulsion between the monomers. The monomers do not dissociate in solution due to the stabilizing effect of the solvent, which maintains finite intermonomer separations.

The O···O distances between the monomers (e.g., 3.771 and 3.800 Å in (ClO_4_^−^)_4_) are significantly longer than twice the van der Waals radius of oxygen (3.1 Å), corresponding to an increase of 22–23%. Such extended distances indicate that direct van der Waals contacts are weak or absent. However, the MESP of the individual monomers overlap in the complexes (Figure 3e,g), suggesting the presence of weak, long-range attractive interactions that contribute to overall stabilization.

We identified only two σ-holes on chlorine in the terminal ClO_4_^−^ units of (ClO_4_^−^)_4_, both exhibiting strongly *V_S,max_* values (*V_S,max_* = 217.6 kcal mol^−1^). The number and magnitude of these extrema increase, as expected, when a higher isodensity envelope is used (e.g., a total of eight extrema, with two on each Cl atom in ClO_4_^−^ on the 0.0015 a.u. isodensity envelope). However, no distinct σ-holes are observed in the intermolecular bonding region either in (ClO_4_^−^)_3_ or in (ClO_4_^−^)_4_, as they are effectively screened and hence quenched due to intermonomer overlap.

Our analyses are supported by the bond paths, critical points, and isosurface topologies between interacting O sites predicted by QTAIM and IGMH, indicating the presence of weak interactions between the monomers (see Figure 3e–h). Although these interactions involve oxygen atoms and display partial directionality, their occurrence between two fully anionic species precludes them as classical chalcogen bonds.

#### 3.3.2. Dimers of BrO_4_^−^

**Figure 4:**
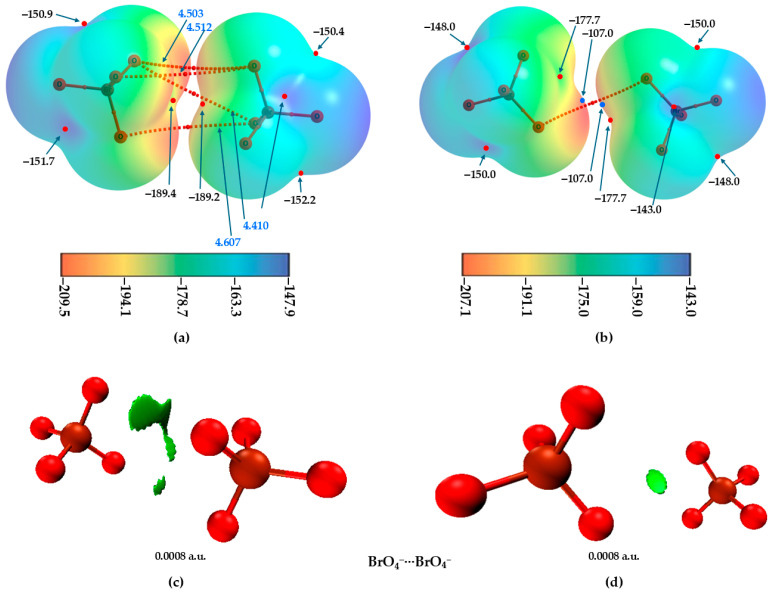
(**a**,**b**) QTAIM molecular graphs superimposed on the MESP maps for two configurations of the (BrO_4_^−^)_2_ dimer computed at the M06-2X/SMD level; the 0.001 a.u. isodensity envelop was used to map the potential. The maxima (tiny red circles) and minima (tiny blue circles) of the electrostatic potential are given in kcal mol^−1^. (**c**,**d**) IGMH isosurface plots for the corresponding configurations shown at an isovalue of 0.0008 a.u.

Two geometries of the (BrO_4_^−^)_2_ dimer were fully optimized starting from the corresponding configurations of (ClO_4_^−^)_2_ shown in Figure 3a,b. The monomers in the (BrO_4_^−^)_2_ dimer in Figure 4a are well separated as observed for (ClO_4_^−^)_2_. However, the Br···Br and O···O distances are 5.343 Å and 4.410–4.607 Å, respectively. These large separations indicate that the monomers are not bonded to each other. Instead, solvation stabilizes the pair at this finite separation, preventing them from drifting to infinity as would be expected in gas-phase calculations dominated by coulombic repulsion. This non-bonded arrangement is further supported by the presence of two maxima of the electrostatic potential within the intermolecular region (*V_S,max_* = −189.4, and 189.2 kcal mol^−1^), each originating from a monomer along the extension of an O–Br bond.

In another configuration of the same (BrO_4_^−^)_2_ dimer shown in Figure 4b, the stoichiometry between the monomeric anions remains essentially the same after energy minimization; however, the anions are separated by O···O and Br···Br distances of 4.568 Å and 5.077 Å, respectively, indicating that attractive interactions between them are negligible. This conclusion is supported by the MESP plot, which shows no overlap between the electrostatic surfaces of the monomers in the optimized complex geometry, although the strength of the maxima of the potential along the extensions of the O–Br bonds are slightly perturbed.

QTAIM analysis reveals four O⋯O bond paths in the first complex and only one in the second, with no bond paths corresponding to Br⋯O, Figure 4a,b. A faint IGMH isosurface volume becomes visible between the O atoms only at a very low isovalue of 0.0008 a.u. (see Figure 4c,d), indicating extremely weak interactions, consistent with the multiple bond-path features predicted by QTAIM for the former conformer. However, the appearance of the isosurface volume only at such low isovalues, together with the absence of any overlap region in the MESP, suggests that the attractive engagement between the interacting monomers may be negligible and that the QTAIM bond paths likely arise from subtle density topology rather than genuine intermolecular interactions.

The SAPT2+3(CCD) analysis of the dimers in the gas phase indicates that the BrO_4_^−^ complexes are more stable than the corresponding ClO_4_^−^ dimer. For the (BrO_4_^−^)_2_ dimer, the total interaction energies are 51.7 kcal mol^−1^ for configuration in Figure 4a and 56.3 kcal mol^−1^ for the configuration in Figure 4b, compared to 69.0 kcal mol^−1^ for (ClO_4_^−^)_2_. In both BrO_4_^−^ dimers, the repulsive electrostatic component dominates the interaction (53.7–59.1 kcal mol^−1^), while induction, and dispersion contributions are comparatively small, indicating that the stability is primarily driven by induction and dispersion. The exchange component is vanishingly small, indicating that the orbital overlap is nearly negligible or very weak.

The binding energies, *E_b_*, of (BrO_4_^−^)_2_ dimers computed directly from SMD total energies are not reported because they are unrealistically large. In continuum solvation, the SMD model artificially stabilizes the dimer relative to the monomers due to charge screening, even when there is no covalent or noncovalent bonding. For the smaller ClO_4_^−^ dimer, the SMD-calculated binding energy is near zero (−0.07 kcal mol^−1^), reflecting the van der Waals type anti-electrostatic interaction between the monomers. The anomalous result for BrO_4_^−^ arises from the interplay of its intermediate size and polarizability: the SMD continuum over-stabilizes the dimer by screening the repulsion between the like-charged anions and allowing partial overlap of their solvation cavities. This artifact is not present for smaller ClO_4_^−^ or larger IO_4_^−^ (see below), where solvation effects do not produce such exaggerated stabilization.

#### 3.3.3. The Dimers of IO_4_^−^

**Figure 5:**
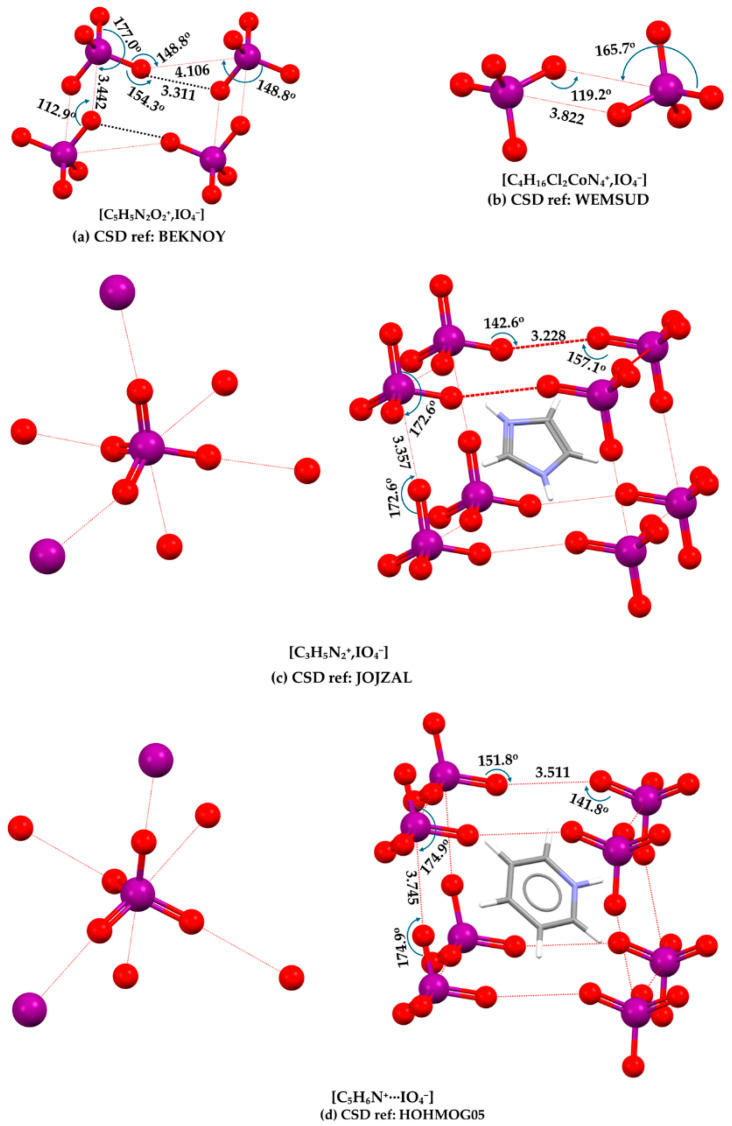
(**a**–**d**) Illustration of the local intermolecular close contacts between periodate anions in selected crystal structures from the CSD. Selected bond lengths (Å) and angles (°) are indicated. Organic cations are omitted in most cases for clarity. The close contacts, including the hanging contacts, are shown as dotted lines in red/black.

The spatial arrangement of the IO_4_^−^ anions varies across different crystal structures. A few (IO_4_^−^)_2_ dimers were extracted from the crystals (CSD refs. BEKNOY, WEMSUD, JOJZAL, and HOHMOG05) and examined using the SMD. In three of these crystal structures, the intermolecular interactions between the anions occur in two distinct forms: O···I and O···O close contacts. In both cases, the separations exceed the sums of the van der Waals radii of the respective atomic basins. Each of the σ-hole regions of O and I in IO_4_^−^ participates in the formation of O···I and O···O close contacts, as illustrated in Figure 5a–d.

In [C_5_H_6_N^+^, IO_4_^−^] (CSD ref: HOHMOG05) and [C_3_H_3_N_2_^+^, IO_4_^−^] (CSD ref: JOJZAL), the O···I contacts generate a two-dimensional architecture, while the involvement of the O···O interactions lead to a three-dimensional cage-like framework in which the organic cation is encapsulated. This is shown in Figure 5c,d, along with the local nature of the close contacts around each anion (see the dangling contacts).

In the other two structures shown in Figure 5a,b, a dimeric arrangement is primarily formed through a pair of equivalent O···I close contacts. Additionally, as illustrated in Figure 5a, a similar contact is observed in an orthogonal direction, although it is long-ranged; the presence of possible O···O close contacts also cannot be overlooked. Both the O···O and O···I contact distances vary significantly from one crystal structure to another and exhibit pronounced directionality.

**Figure 6:**
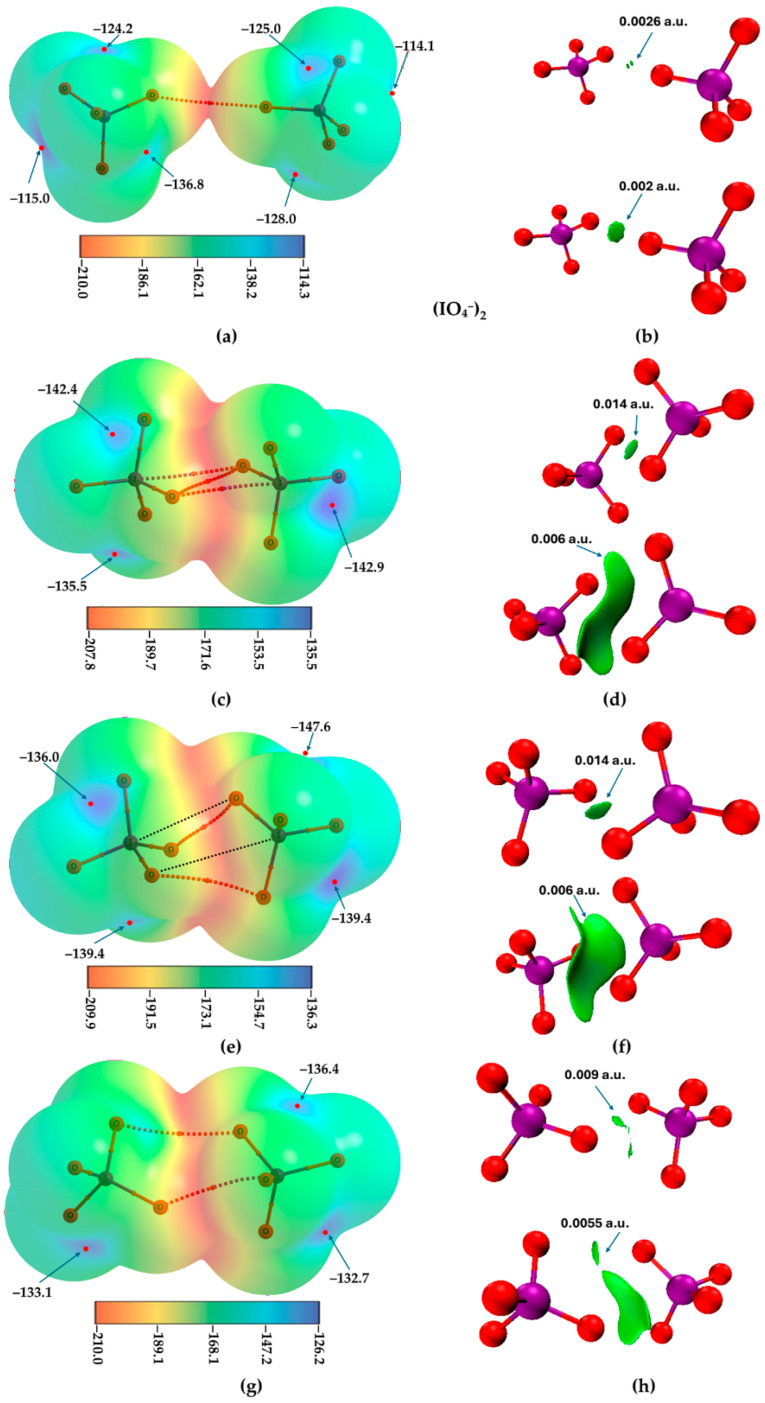
(**a**,**c**,**e**,**g**) QTAIM molecular graphs superimposed on MESP surfaces for (IO_4_^−^)_2_ dimers. (**b**,**d**,**f**,**h**) Corresponding IGMH isosurface representations. σ-Holes (highlighted by red circles) are indicated with arrows, and the corresponding *V_S,max_* values (kcal mol^−1^) are shown. The IGMH isovalues used for each system are specified in the respective panels.

Nevertheless, the O···O distance in the (IO_4_^−^)_2_ dimer shown in Conf1 (Figure 6a) is 3.766 Å in the energy-minimized geometry (3.511 Å in the corresponding crystal structure; Figure 5d), with an I–O···O angle of 160.4° (158.8° in the crystal). The O···O separation is some 21% longer than twice the van der Waals radius of oxygen (*r*_vdw_(O) = 1.55 Å), and about 7% longer than in the crystal structure. Even accounting for ±0.2 Å uncertainty in *r*_vdw_(O), the contact remains longer than 3.766 Å, suggesting that the interaction is not stabilizing.

QTAIM predicts a bond path and bond critical point between the two O atomic basins of the dimer (*ρ*_b_ = 0.0013 and ∇^2^*ρ*_b_ = 0.0050 a.u.) (Figure 6a), and IGMH reveals a weak isosurface that appears only at an isovalue of 0.002 a.u. (Figure 6b). The binding energy of the dimer is small and positive (*E_b_* = +0.54 kcal mol^−1^), consistent with a van der Waals–type contact. Clearly, the O···O close contact in (IO_4_^−^)_2_ should not be regarded as a chalcogen bond, but rather as an unconventional close contact, stabilized by the medium that keeps the monomers in proximity. In the gas phase, the monomers repel each other.

Application of the PCM model further separates the two monomers in (IO_4_^−^)_2_, resulting in an O···O distance of 3.911 Å and an O···O–I (I–O···O) angle of 160.3° (142.4°), indicating increased repulsion compared to the M06-2X/SMD-optimized geometry (vide supra). The corresponding binding energy is +0.44 kcal mol^−1^.

The σ-holes along the I–O bond extensions on the interacting oxygen atoms are absent in the dimer, reflecting their annihilation at the equilibrium geometry. This is consistent with overlapping molecular electrostatic potentials of the monomers, indicative of a weak intermolecular contact. However, the iodine atom retains all four non-interacting σ-holes on the 0.001 a.u. electron density isosurface of the (IO_4_^−^)_2_ complex. The *V_S,max_* values are −115.0, −124.2, −124.9, and −136.8 kcal mol^−1^ for one iodine atom, and −125.0, −128.0, −130.1, and −114.1 kcal mol^−1^ for the other. The corresponding values obtained using the PCM model are −119.2, −128.4, −128.9, and −140.8 kcal mol^−1^ for one iodine atom, and −118.5, −129.5, −132.0, and −134.3 kcal mol^−1^ for the other.

QTAIM analysis reveals three topological features between the interacting monomers for Conf2, a dimer that was extracted from the crystal structure with CSD ref. BEKNOY (Figure 6c). Two of these are associated with O···I close contacts, while the third corresponds to an O···O close contact. The first two are equivalent, with *r*(O···I) = 3.233 Å, ∠I···O–I = 109.0°, and ∠O–I···O = 179.5°, the latter angle suggesting a σ-hole-centered interaction at iodine. By contrast, the O···O close contact is characterized by *r*(O···O) = 3.139 Å and ∠O···O–I = 76.9°. The O···I interactions follow a Type-II topology, whereas the O···O interaction corresponds to a Type-I topology of chemical interactions. The former interaction leads to the annihilation of two σ-holes on the iodine atoms, while three non-interacting σ-holes remain on each iodine, with *V_S,max_* values of −135.5, −142.4, and −142.9 kcal mol^−1^. The IGMH isosurface plot in Figure 6d indicates that the O···O contact is primary (top panel), whereas the O···I interaction is secondary, as the former appears at higher isovalue of 0.014 a.u. and the latter only becomes evident at an isovalue below 0.009 a.u. (bottom panel).

The molecular graph of Conf3, Figure 6e, extracted from the crystal structure (CSD ref. BEKPAM), shows two equivalent bond paths between the oxygen atomic basins, with *r* = 3.156 Å and ∠I–O···O = 133.1°. However, based on the geometric arrangement, additional bond paths between O and I would be expected, giving rise to two equivalent O···I close contacts. This expectation is supported by the intermolecular parameters, with *r* = 3.246 Å, ∠O–I···O = 178.0°, and ∠I···O–I = 105.4° for each contact. The complex has three negative σ-holes on iodine in each IO_4_^−^ monomer (*V_S,max_* = −136.0, −139.4, and −147.6 kcal mol^−1^), while the remaining σ-hole is effectively quenched upon dimer formation. Additionally, there may be further O···O close contacts within a separation distance of approximately 3.3 Å. All these close contacts between the monomers, which are neither halogen bonds nor chalcogen bonds, are reflected in the IGMH isosurface topology, which appears prominent at an isovalue of 0.006 a.u. (Figure 6f, bottom) However, as indicated above, the primary interaction is associated with the O···O contact, as evidenced by the presence of a more localized and irregular isosurface volume that persists at a higher isovalue of 0.014 a.u. (Figure 6f, top).

The QTAIM molecular graph shown in Figure 6g for Conf4 (extracted from CSD ref. JOJZOL) reveals two bond paths corresponding to O···O and I···O close contacts. The former corresponds to a Type-I interaction and is the longest (*r* = 3.473 Å, ∠I–O···O = 128.4°, ∠O···I–O = 96.8°), whereas the latter corresponds to a Type-II interaction and is shorter (*r* = 3.409 Å, ∠O–I···O = 179.2°). However, QTAIM does not identify additional O···O close contacts present at separation distances of 3.302, 3.266, and 3.296 Å. Nevertheless, the iodine atom in one monomer exhibits three σ-holes (*V_S,max_* = −132.7, −136.3, and −136.4 kcal mol^−1^), whereas the same atom in the other monomer exhibits four (*V_S,max_* = −126.2, −133.1, −151.7, and −153.4 kcal mol^−1^). The absence of a σ-hole on an iodine atom may be attributed to its involvement in the formation of an I···O close contact. This interpretation is also consistent with the IGMH isosurface shown in Figure 6h (top), where a faint feature at an isovalue of 0.009 a.u. corresponds to the O···I contact. The full extent of the interactions, including both O···O and O···I contacts, becomes evident at a lower isovalue of 0.0055 a.u. (see Figure 6h, bottom).

The energy stability trend (from most to least stable) is: Conf3 (−2.02 kcal mol^−1^) > Conf2 (−1.65 kcal mol^−1^) > Conf4 (−0.44 kcal mol^−1^) > Conf1 (0.54 kcal mol^−1^).

#### 3.3.4. The Oligomers of IO_4_^−^

**Figure 7:**
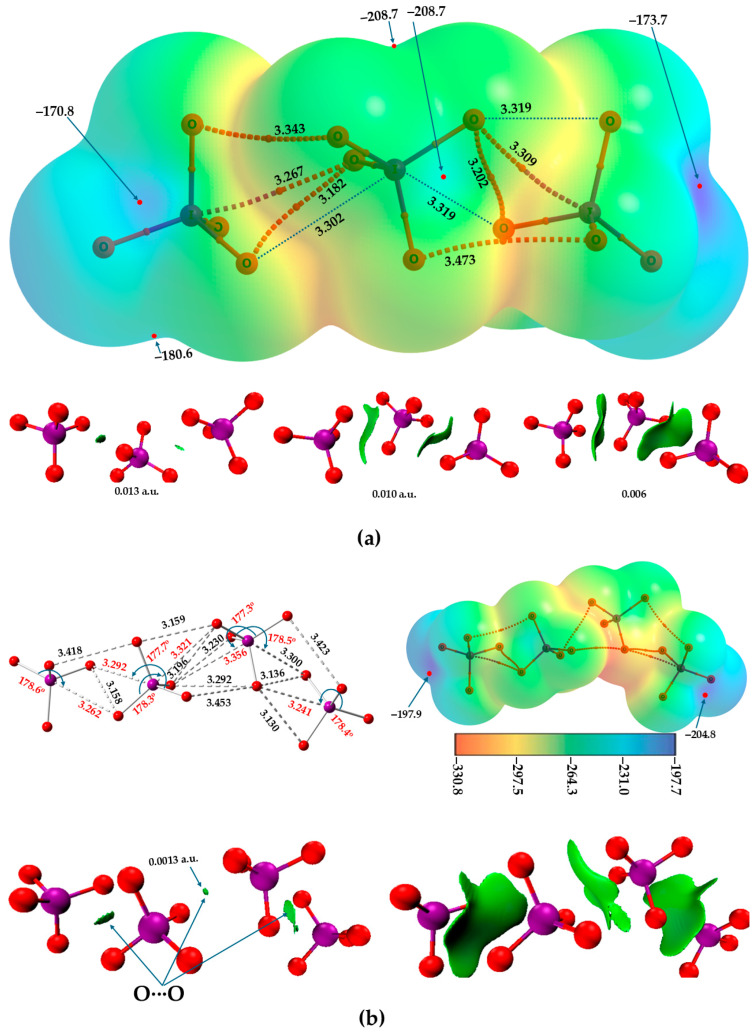
(**a**) (**top**) QTAIM molecular graph superimposed on the MESP plot and (**bottom**) IGMH isosurface plot of (IO_4_^−^)_3_ obtained from the SMD-optimized geometry. (**b**) (**top**-**left**) ball-and-stick representation, (**top**-**right**) QTAIM molecular graph superimposed on the MESP plot, and (**bottom**) IGMH isosurface plot of (IO_4_^−^)_4_ obtained from the SMD-optimized geometry. Selected bond distances in ((**a**), **top**) and ((**b**), **top**-**left**) are given in Å, and the maxima of the electrostatic potential (small red circles indicated by arrows) are reported in kcal mol^−1^.

The (IO_4_^−^)_3_ trimer was also examined. We found that the iodine atom in the terminal monomer exhibits three σ-holes, whereas that in the central monomer has only two instead of four, which are all negatively strengthened upon the formation of intermolecular interactions (see Figure 1c vs. Figure 7a). This absence of a σ-hole in the terminal monomers suggests its possible involvement in attractive interactions with neighboring oxygen atoms in adjacent monomers, facilitating the formation of close O⋯I close contacts. QTAIM analysis of the bond paths and bond critical points confirms a pair of O⋯I contacts and indicates the presence of O⋯O close contacts that are slightly longer than twice the van der Waals radius of oxygen (3.1 Å). Careful inspection of the trimer geometry shows O⋯I bond distances and the corresponding ∠O–I⋯O angles as 3.267 Å (178.5°), 3.302 Å (178.9°), 3.319 Å (178.9°), and 3.309 Å (179.2°), demonstrating that QTAIM failed to identify two of these I’s σ-hole centered O⋯I contacts that are marked by dotted lines in black (Figure 7a).

The IGMH analysis supports these conclusions and shows that the primary interactions between the anions are O···O contacts, which persist at an isovalue of 0.014 a.u. but disappear at 0.015 a.u. (Figure 7a, bottom). By contrast, O···I contacts emerge below 0.012 a.u. and become clearly visible at isovalues < 0.010 a.u. The isosurface volumes shown in Figure 7a (bottom), computed at 0.013, 0.010, and 0.006 a.u., confirm the presence of both O···O and O···I interactions in (IO_4_^−^)_3_.

The bonding interactions revealed by QTAIM for (IO_4_^−^)_4_, (Figure 7b, top-right), do not fully correspond to those inferred from intermolecular distances (Figure 7b, top-left). For instance, no bonding topology is identified for an O···O contact at 3.292 Å, whereas it is predicted for distances of 3.158 and 3.418 Å between the first two IO_4_^−^ units (from left). A comparable trend is observed for the last two IO_4_^−^ units, where O···O contacts at 3.136 and 3.423 Å are identified, and are not chalcogen bonds. Between the intermediate units, QTAIM predicts bond paths for O···O separations of 3.196 and 3.453 Å, despite several other close contacts falling within a similar distance range. Likewise, although multiple O···I close contacts exist among the four IO_4_^−^ units, QTAIM reveals only two of them in the (IO_4_^−^)_4_ tetramer with *r* < 3.263 Å, but not those with slightly longer separations (*r* < 3.36 Å). These latter interactions are directional and centered on negative σ-holes on I, and therefore do not correspond to conventional halogen bonds.

The inconsistent appearance of bond paths for comparable intermolecular separations suggests a degree of arbitrariness in the QTAIM description, indicating that the presence or absence of a bonding topology is not always a reliable indicator of the underlying weak interactions.

IGMH analysis shows that the O···O close contacts in (IO_4_^−^)_4_ associated with the shortest distances remain dominant even at an isovalue of 0.013 a.u., where all other intermolecular interactions disappear (see Figure 7b, bottom-left). At a lower isovalue of 0.006 a.u., all interactions remain visible as green isosurface volumes (Figure 7b, bottom-right), revealing that some of the secondary O···O close contacts are weaker than the highly directional O···I close contacts.

The overall binding energy of (XO_4_^−^)_n_ (*n* = 2–4) was computed from the total energies of the identical monomeric units as defined in Equation (1), where *E* represents total electronic energy of respective species in solution. This yields $E_{b}^{o}$ values of −3.27 and −4.13 kcal mol^−1^ for the trimer and tetramer, respectively.

(1) $$E_{b}^{o}=E(\left({XO}_{4}^{-}{)}_{n}\right)-n\;E\left({XO}_{4}^{-}\right)$$

The stepwise binding energy, corresponding to the addition of one monomer to form the trimer and tetramer from the dimer and trimer, respectively, was calculated using Equation (2), where *n* = 3 and 4.

(2) $$E_{b}^{s}=E(({XO}_{4}^{-}{)}_{n})-E(({XO}_{4}^{-}{)}_{n-1})-E({XO}_{4}^{-})$$

This gives $E_{b}^{s}$ values of −1.62 and −0.86 kcal mol^−1^ for the formation of the trimer (IO_4_^−^)_3_ and tetramer (IO_4_^−^)_4_, respectively. Comparison with the stepwise binding energy of the dimer (−1.65 kcal mol^−1^) indicates weak negative cooperativity, as the addition of successive monomers becomes progressively less stabilizing from dimer to trimer to tetramer.

By contrast, oligomers of ClO_4_^−^ exhibit markedly different behavior. While the dimer is only weakly stabilized (*E_b_* = −0.08 kcal mol^−1^), the trimer and tetramer are progressively destabilized, with overall binding energies of +0.75 and +1.97 kcal mol^−1^, respectively. The corresponding stepwise energies further confirm this trend, showing a small stabilization for dimer formation followed by increasing energetic penalties for subsequent monomer additions (+0.83 kcal mol^−1^ for the third unit and +1.22 kcal mol^−1^ for the fourth), indicative of strong anti-cooperative behavior.

#### 3.3.5. Isovalue Sensitivity of IGMH Isosurfaces in Probing Weak Anion–Anion Interactions

**Figure 8:**
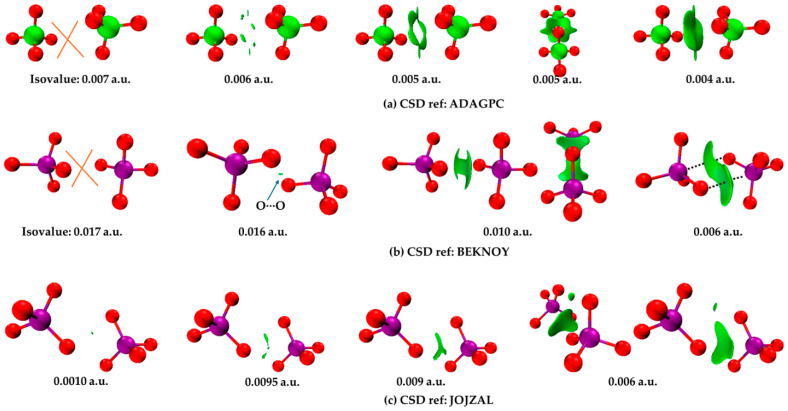
Dependence of IGMH isosurface features on the chosen isovalue in revealing the presence and relative strength of intermolecular interactions in (**a**) (ClO_4_^−^)_2_, (**b**) (IO_4_^−^)_2_, and (**c**) (IO_4_^−^)_2_ dimers. Green regions between monomers represent IGMH isosurface volumes, with the corresponding isovalues indicated in each panel. Red crosses in (**a**,**b**) denote the absence of detectable isosurfaces at the specified isovalues. The dimers were obtained from energy-minimized structures extracted from the corresponding CSD crystal geometries.

As shown in Figure 8a, no discernible IGMH isosurface is observed between the ClO_4_^−^ anions in (ClO_4_^−^)_2_ at an isovalue of 0.007 a.u.; however, faint features emerge upon lowering the isovalue to 0.005–0.006 a.u., confirming the extremely weak nature of the interaction. A clearer, though still diffuse, isosurface is obtained at 0.004 a.u., indicating that the interaction becomes appreciable only at low isovalues.

The interaction between the anions in the dimer is not purely σ-hole-centered but instead arises from multiple weak O···O contacts, in which each oxygen atom of one monomer interacts with neighboring oxygen sites of the other. This is consistent with isosurface features observed at isovalues of 0.0045–0.005 a.u.

For (IO_4_^−^)_2_ (Figure 8b), the same conformer shown in Figure 6c, the isosurface disappears at relatively high isovalues but begins to emerge upon decreasing the isovalue to 0.0016 a.u., where a small feature suggests a weak O···O interaction. As the isovalue is lowered further to 0.010 a.u., the isosurfaces become more pronounced, indicating enhanced interaction visibility. The green, relatively flat isosurface between the molecular entities indicates a weak but attractive interaction, while more pronounced features between the O and I atomic basins point to stronger O···I contributions. Given the I···I separation of 4.155 Å, weak interactions between the iodine centers may also be expected. Whether this should be considered stabilizing or destabilizing remains an open question.

In the configuration shown in Figure 6c, which is the same as that shown as Figure 6g, no intermolecular isosurfaces are observed at an isovalue of 0.011 a.u., while a faint O···O feature appears at 0.010 a.u. Upon further reduction in the isovalue, additional interactions, including O···I contacts, begin to develop. At approximately 0.006 a.u., a semi-elliptical isosurface between oxygen atoms becomes evident, indicating another weak O···O interaction. The emergence of these features only at relatively low isovalues confirms the weak nature of the interactions and suggests that this dimer is less stable than the one discussed above, consistent with the corresponding binding energies (*E_b_* = −1.65 kcal mol^−1^ versus ≈ −0.44 kcal mol^−1^). These results suggest that the primary or secondary character of an interaction in IGMH analysis is largely geometry dependent. Shorter interatomic distances lead to stronger isosurface features, and vice versa.

### 3.4. The Origin of Interaction: Symmetry Adapted Perturbation Theory Analysis

Across the halate complexes, the SAPT analysis confirms that electrostatics is the dominant repulsive contribution in all cases, ranging from ~48.9 kcal mol^−1^ in IO_4_^−^···IO_4_^−^ to ~73.8 kcal mol^−1^ in (ClO_4_^−^)_2_ (see Table 2). The chlorine-containing complex exhibits the largest total interaction energy (69.0 kcal mol^−1^), driven primarily by its strong electrostatic component, while bromate systems are comparatively weaker.

Induction is consistently stabilizing across all complexes, with relatively small contributions in BrO_4_^−^ systems (−1.6 to −2.1 kcal mol^−1^). This is arguably a result of minimal orbital overlap between interacting domains, as revealed by the low exchange repulsion components. As can be seen from Table 2, induction energy is smaller for these systems relative to the significantly larger values predicted for the iodine-based systems, particularly IO_4_^−^···IO_4_^−^ (JOJZAL; Figure 6g) (−4.3 kcal mol^−1^) that has an O···I close contact and IO_4_^−^···IO_4_^−^ (BEKPAM; Figure 6e) (−5.7 kcal mol^−1^), as well as in the ClO_4_^−^ complex (−4.0 kcal mol^−1^), indicating enhanced polarization effects. For example, exchange repulsion is minimal in the BrO_4_^−^ systems (0.0–0.1 kcal mol^−1^) but increases markedly in iodine-containing complexes, particularly IO_4_^−^···IO_4_^−^ (JOJZAL) (4.3 kcal mol^−1^) and even more so in IO_4_^−^···IO_4_^−^ (BEKPAM) (13.3 kcal mol^−1^), reflecting stronger electronic overlap and steric effects.

Dispersion is also stabilizing in all cases and becomes especially important for the heavier and more polarizable systems, reaching −7.9 kcal mol^−1^ in IO_4_^−^···IO_4_^−^ (BEKPAM), compared to only −0.5 to −0.8 kcal mol^−1^ in BrO_4_^−^ complexes.

The interaction strength follows the trend: (ClO_4_^−^)_2_ (ADAGPC) < IO_4_^−^···IO_4_^−^ (BEKPAM) < IO_4_^−^···IO_4_^−^ (JOJZAL) < (BrO_4_^−^)_2_ (ADAGPC) < (BrO_4_^−^)_2_ (ADAWAG) < IO_4_^−^···IO_4_^−^ (HOHMOG05).

### 3.5. Solvent-Mediated Attraction Between Electron-Rich Sites in Neutral Molecules

The solution-phase calculations suggest a similar distribution of electrostatic potential on the surfaces of molecules such as CO, N_2_, and CH_3_F that might be expected in the gas phase. Accordingly, a maximum of potential is observed on F (−28.6 kcal mol^−1^) along the C–F bond extension in CH_3_F, which is slightly more negative than that obtained in the gas phase (see above). Similarly, the C- and O-ends of CO exhibit negative minima of potential along the C–O bond extension (*V_S,min_* = −12.9 and −7.2 kcal mol^−1^, respectively). For N_2_ and HCN, the corresponding minima of potential on N along the outer extensions of the N≡N and C≡N bonds are −8.6 and −41.1 kcal mol^−1^, respectively, whereas that on O along the C=O bond extension in H_2_CO is −41.3 kcal mol^−1^.

**Figure 9:**
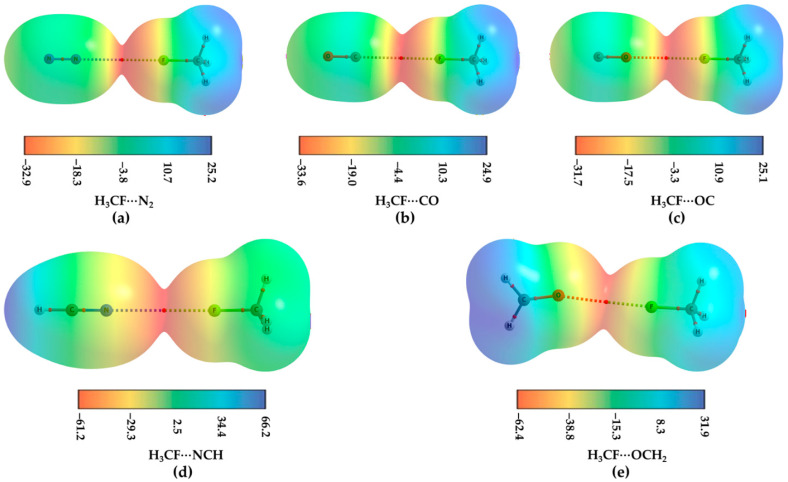
(**a**–**e**) QTAIM molecular graphs superimposed on MESP plots of selected CH_3_F dimers, illustrating attractive interactions between electron-rich sites driven by the solvated medium. Electrostatic potential values are given in kcal mol^−1^. Bond paths are shown as solid and dashed lines in atom colors, and bond critical points are represented as small red spheres along the bond paths.

The solution phase can stabilize the interacting monomers in such a way that it can enable attractive interactions between the negative sites that would otherwise be repulsive in the gas phase. For example, we considered neutral systems in which the negative σ-hole on F along the C–F bond extension in CH_3_F interacts attractively with the lone pair on N along the extension of N_2_ (Figure 9a). A similar behavior is observed when the C- and O-ends of CO, N-end of HCN, and O-end of H_2_CO are brought into proximity with the F atom of CH_3_F, leading to dimer formation (Figure 9b–e). As noted above, such spatial arrangements would be repulsive in the gas phase based on Coulomb’s law of electrostatics. Nevertheless, while the close contacts are directional with respect to the negative σ-hole on F along the C–F bond extension, these are neither halogen bonds nor pnictogen or tetrel bonds; rather, they are best described as solvent-medicated anti-electrostatic interactions based on SAPT (vide infra).

In all cases, a bond path and a bond critical point are identified between the interacting atomic basins, with clear overlap of the molecular surfaces involving the interacting entities, confirming the presence of intermolecular interactions between the monomers. The intermolecular distances (and angles) for the C–F···C, C–F···O, C–F···N, C–F···N, C–F···N, and C–F···O, contacts are 3.438 Å (180.0°), 3.194 Å (180.0°), 3.562 (180.0°), 3.587 (179.1°), and 3.243 Å (174.3°) for H_3_CF⋯CO, H_3_CF⋯OC, H_3_CF⋯N_2_, H_3_CF⋯NCH, and H_3_CF⋯OCH_2_, respectively. The charge density (and the Laplacian of the charge density) at the corresponding bcps is 0.0028 (0.0119), 0.0027 (0.0150), 0.0015 (0.0072), 0.0017 (0.0076), and 0.0027 (0.0139) a.u., respectively, suggesting closed shell interactions.

Table 3 shows that all complexes reflect a balance between stabilizing dispersion and induction and destabilizing electrostatics and exchange, obtained using fully relaxed SMD geometries of the dimers in the gas phase. In every case, dispersion is the dominant attractive contribution, while electrostatics largely dictates whether the overall interaction is favorable or not. H_3_CF⋯OC remains the only net stabilized system (Total SAPT2+3(CCD) = −0.04 kcal mol^−1^), where dispersion (−0.41 kcal mol^−1^) slightly outweighs electrostatic (0.19 kcal mol^−1^) and exchange (0.24 kcal mol^−1^) penalties. Both H_3_CF⋯CO (+0.27 kcal mol^−1^) and H_3_CF⋯N_2_ (+0.05 kcal mol^−1^) are weakly destabilized; the complex with CO shows stronger electrostatic and exchange repulsion despite having the largest dispersion, while that with N_2_ has reduced dispersion due to its lower polarizability.

For H_3_CF⋯NCH and H_2_CF⋯OCH_2_, the interaction trends become more pronounced and consistent. The strongest destabilization is observed for H_3_CF⋯NCH (+1.10 kcal mol^−1^) and H_2_CF⋯OCH_2_ (+1.08 kcal mol^−1^), both of which exhibit very large electrostatic repulsion (1.37–1.41 kcal mol^−1^). Although these systems also benefit from moderate dispersion (−0.30 to −0.43 kcal mol^−1^) and induction (−0.09 to −0.12 kcal mol^−1^), these stabilizing effects are insufficient to overcome the strong repulsion, indicating unfavorable orientations.

The binding energies *E_b_* reveal a complementary story: all complexes have negative *E_b_* values, indicating that they are bound when computed with conventional supermolecular approaches. The strongest binding is seen for H_3_CF⋯CO (−0.55 kcal mol^−1^) and H_3_CF⋯OC (−0.44 kcal mol^−1^), followed by H_2_CF⋯OCH_2_ (−0.37 kcal mol^−1^), H_3_CF⋯N_2_ (−0.23 kcal mol^−1^), and H_3_CF⋯NCH (−0.21 kcal mol^−1^). This apparent discrepancy with SAPT totals highlights that different energy decomposition schemes can emphasize different aspects of the interaction, especially in very weak, dispersion-dominated systems. The data show that while dispersion consistently stabilizes all complexes, electrostatic repulsion controls the SAPT energy trends, whereas *E_b_* suggests all systems are weakly bound, with stability decreasing in the order H_3_CF⋯CO > H_3_CF⋯OC > H_2_CF⋯OCH_2_ > H_3_CF⋯N_2_ > H_3_CF⋯NCH.

The dissociation of the dimers in the gas phase suggests that the interaction lacks intrinsic stability and is better described as a weak, environment-stabilized association rather than a genuine intermolecular bond.

## 4. Influence of the Periodic Crystal Environment on Anion–Anion Interactions

To further assess the intrinsic stability of isolated anion–anion pairs, geometry optimizations were performed for the (ClO_4_^−^)_2_ and (BrO_4_^−^)_2_ dimers in a large cubic vacuum cell (*a* ≈ 30 Å). In both cases, the anions do not retain compact configurations upon relaxation and instead separate significantly during optimization, consistent with strongly repulsive interactions in the absence of a periodic crystal environment. This behavior is independent of the halogen identity and is similar to that observed in gas-phase calculations, indicating that isolated anion–anion assemblies are not intrinsically stable without crystal packing and electrostatic stabilization.

To directly evaluate the role of the crystal environment in stabilizing the short anion–anion contacts, periodic DFT calculations were additionally performed on a representative crystal structure containing perchlorate and perbromate anions. Single-crystal X-ray diffraction analysis revealed that the compound triaqua-tin(II) diperchlorate, [Sn(H_2_O)_3_]^2+^·2(ClO_4_^−^) (CSD ref: FOSROW), crystallizes in the hexagonal space group *P*6_3_ with Z = 2 and Z′ = 0.333333. The unit-cell parameters are *a* = *b* = 7.0701(10) Å and *c* = 9.7631(15) Å, with α = β = 90° and γ = 120°. The final refinement converged with an R-factor of 3.64%, indicating good agreement between the experimental diffraction data and the refined structural model.

Periodic geometry optimizations were carried out using both fixed-cell (ISIF = 2) and fully relaxed (ISIF = 3) conditions. In both cases, the experimentally observed crystal packing arrangement and the short perchlorate–perchlorate contacts were retained. Full relaxation produced only a modest lattice expansion (*a* = *b* = 7.121 Å, *c* = 9.821 Å) while preserving the hexagonal symmetry (space group *P*6_3_) and the overall crystal packing motif. No structural collapse, proton transfer, or chemical rearrangement was observed during the periodic optimizations.

**Figure 10:**
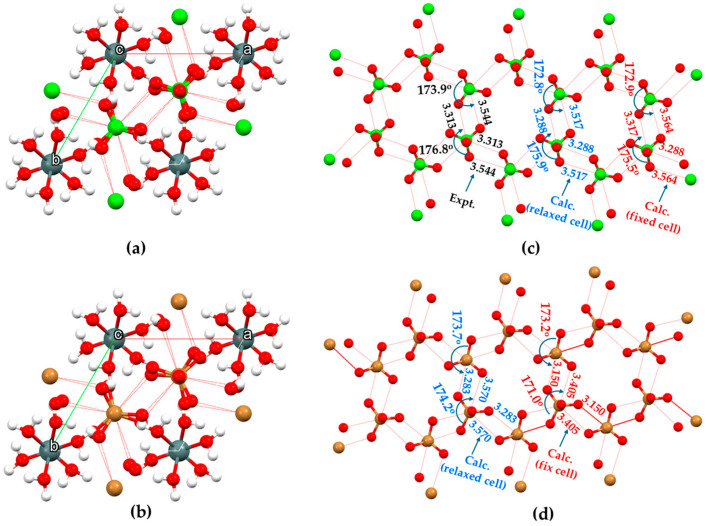
The PBE-level optimized unit-cell geometries of (**a**) [Sn(H_2_O)_3_]^2+^·2(ClO_4_^−^) and (**b**) [Sn(H_2_O)_3_]^2+^·2(BrO_4_^−^), with the lattice vectors shown. Panels (**c**,**d**) show a comparison of the crystallographic and calculated local anion–anion interaction topologies observed in the corresponding ClO_4_^−^- and BrO_4_^−^-containing lattices, respectively. Selected Cl···O separations and O–Cl···O angles are given in Å and degrees (°), respectively. The unit cells in (**a**,**b**) are viewed along the crystallographic *c*-axis. Close contacts are indicated by dashed red lines.

To further investigate the influence of anion substitution within the same crystal environment, the experimentally derived lattice of [Sn(H_2_O)_3_]^2+^·2(ClO_4_^−^) was used to construct the corresponding bromate analogue, [Sn(H_2_O)_3_]^2+^·2(BrO_4_^−^) by replacing Cl with Br while retaining the same initial crystal framework. Periodic geometry optimizations were subsequently performed under both fixed-cell and fully relaxed conditions. In contrast to the perchlorate system, full relaxation of the bromate analogue resulted in a substantially larger lattice expansion accompanied by symmetry lowering from hexagonal *P6_3_* to a distorted hexagonal lattice exhibiting monoclinic *P2_1_* symmetry. The optimized lattice parameters increased to *a* = *b* = 7.228 Å and *c* = 10.240 Å, accompanied by an increase in the unit-cell volume from 422.639 to 463.281 Å^3^. These results indicate that the larger BrO_4_^−^ anion is less compatible with the original crystal packing arrangement, emphasizing the important role of the surrounding periodic crystal environment in stabilizing the experimentally observed short anion–anion contacts. A detailed comparison of lattice parameters is provided in Table 4, while Figure 10a–d illustrates the differences between the experimental crystal structures and the corresponding optimized geometries obtained from periodic DFT calculations.

The packing in the crystal of [Sn(H_2_O)_3_]^2+^·2(ClO_4_^−^) features a pseudo 2D anionic hexagonal framework exhibiting a distorted Kagome-like topology (Figure 10b) [40], generated solely through directional σ-hole-mediated Cl···O interactions between $ClO_{4}^{-}$ tetrahedra. Each tetrahedral unit participates in six such interactions with three neighboring anions, forming three pairs of inequivalent Cl···O close contacts. Intermolecular Cl···O contacts between neighboring perchlorate anions generate a corrugated two-dimensional network containing distorted hexagonal motifs that extend along the *a*- and *b*-crystallographic directions, thereby producing an extended grid-like framework. The inequivalent Cl···O separations (and corresponding O–Cl···O angles) are 3.313 and 3.544 Å (176.8° and 173.9°), respectively, in the crystal structure, compared to 3.288 and 3.517 Å (175.9° and 172.8°) in the fully relaxed geometry. In the fixed-cell (ISIF = 2) optimization, the corresponding values are 3.317 and 3.564 Å (175.5° and 172.9°), respectively. The intermolecular interaction features are very similar to those observed for the BrO_4_^−^ anions in the crystal structure of [Sn(H_2_O)_3_]^2+^·2(BrO_4_^−^), as shown in Figure 10c. These results indicate substantial retention of interaction geometry and directionality upon periodic optimization. The framework displays layered hexagonal motifs reminiscent of those observed in ice Ih and some silicate-derived architectures.

**Table 4:** Comparison of experimental and periodic PBE-optimized crystallographic parameters for [Sn(H_2_O)_3_]^2+^·2(ClO_4_^−^) and the corresponding substituted [Sn(H_2_O)_3_]^2+^·2(BrO_4_^−^) system obtained under fixed-cell (ISIF = 2) and fully relaxed (ISIF = 3) periodic boundary condition calculations. The fully relaxed BrO_4_^−^ analogue exhibits increased lattice expansion and symmetry lowering relative to the experimentally observed perchlorate crystal framework ^a^.

Parameter	Experimental ^b^	ClO_4_^−^ (ISIF = 2)	ClO_4_^−^ (ISIF = 3)	BrO_4_^−^ (ISIF = 2)	BrO_4_^−^ (ISIF = 3)
Formula	[Sn(H_2_O)_3_]^2+^·2(ClO_4_^−^)	Sn_2_O_22_H_12_Cl_4_	Sn_2_O_22_H_12_Cl_4_	Sn_2_O_22_H_12_Br_4_	Sn_2_O_22_H_12_Br_4_
Crystal system	Hexagonal	Hexagonal	Hexagonal	Hexagonal	Monoclinic
Space group	*P6*_3_	*P6*_3_	*P6*_3_	*P6*_3_	*P2*_1_
Space group number	173	173	173	173	4
*a* (Å)	7.0701(10)	7.070	7.121	7.070	7.228
*b* (Å)	7.0701(10)	7.070	7.121	7.070	7.228
*c* (Å)	9.7631(15)	9.763	9.821	9.763	10.240
α (°)	90	90	90	90	90
β (°)	90	90	90	90	90
γ (°)	120	120	120	120	120
Cell volume (Å^3^)	422.639	422.639	431.322	422.639	463.281
Density (g cm^−3^)	—	2.920	2.862	3.619	3.301
Symmetry retained	Yes	Yes	Yes	Yes	No
Space-group change upon relaxation	—	None	None	None	*P6_3_* → *P2_1_*

^a^ ISIF = 2: relaxation of ionic positions with fixed lattice parameters; ISIF = 3: simultaneous relaxation of ionic positions, cell shape, and cell volume. ^b^ CSD reference for the crystal is FOSROW [41].

Comparison of the fixed-cell (ISIF = 2) and fully relaxed (ISIF = 3) periodic calculations further highlights the different structural responses of the perchlorate and bromate systems within the crystal environment. For the experimentally observed [Sn(H_2_O)_3_]^2+^·2(ClO_4_^−^) crystal, full relaxation produced only a small additional stabilization of approximately 0.040 eV relative to the fixed-cell optimization, indicating that the experimental crystal packing arrangement is already close to an energetic minimum within the periodic environment. The corresponding [Sn(H_2_O)_3_]^2+^·2(BrO_4_^−^) analogue exhibited a substantially larger stabilization of approximately 0.512 eV upon full relaxation, accompanied by significant lattice expansion and symmetry lowering. The markedly different relaxation behavior between the two systems demonstrates that the stability and geometry of the short anion–anion contacts are strongly dependent on the surrounding crystal packing environment and are highly sensitive to anion substitution and ionic size effects.

## 5. Discussion

The electrophilic character of σ-holes on covalently bonded halogen atoms in molecular entities is well established in the gas phase, where they engage with nearby negative sites and are widely used to rationalize intermolecular interactions governing crystal packing, thereby underpinning classical halogen bonding. However, even when the σ-hole region is negative (electron-rich), as on F in H_3_CF, directional intermolecular interactions can still arise in the gas phase, as well as in solution and strongly polar environments. These interactions do not conform to the classical definition of halogen bonding and are better described as unconventional halogen-centered, “anti-electrostatic” noncovalent interactions.

We have shown that intrinsic gas-phase interactions between XO_4_^−^ anions, as observed in the crystal structures, are not stable in isolation, as electrostatic repulsion drives the system toward complete dissociation. This behavior changes when the systems are modeled in a solvent environment, regardless of whether PCM or SMD solvation models are employed.

The solvent model somehow reproduces dimer and oligomer geometries that are not broadly inconsistent with those observed in infinite crystal. However, it fails to preserve the long-range ordering of molecular entities in linear chain–like arrangements, highlighting the absence of explicit crystal packing forces in the model. This limitation leads to noticeable deviations in intermolecular distances and angles, which are either underestimated or overestimated relative to the crystalline structure. These observations reveal the inherent limitations of implicit solvent models in capturing the collective and cooperative effects that govern structural organization in the crystalline phase.

When dissected energy components of the dimers were evaluated using solution-phase geometries, the resulting interaction energies remain overall positive despite the presence of well-organized X···O or O···O close contacts. This leads to the conclusion that the observed structural motifs in dimers and oligomers arise only when environmental effects—particularly solvent screening, polarization, and dispersion contributions—are included. Clearly, the stability of the inorganic lattice in the solid state should therefore be understood as being driven by crystal packing forces, together with counter ion effects that include attractive interactions of different flavors, that sufficiently attenuate coulombic repulsion, allowing weak attractive interactions between the anions to shape the final geometry. These results indicate that the previously reported [4,10,11] anion–anion X···O interactions in halogen oxyanion solids do not represent intrinsic halogen bonding, but instead reflect environment-assisted, anti-electrostatic associations governed by a delicate balance of noncovalent forces and stabilized indirectly through counterion-mediated packing interactions and other primary intermolecular forces.

Our views are supported by MESP analysis, which shows that the electrostatic surface of the covalently bonded halogen or oxygen center remains negative both in the monomers and dimers and becomes even more negative in the oligomers. Consequently, the σ-hole-centered interactions offered by the halogen derivative or oxygen cannot be classified as halogen bonds, or chalcogen bonds.

The assignment of bonding interactions by QTAIM is not always reliable for weak and diffuse interactions. In particular, it has failed to identify certain interactions or inconsistently capture close contacts of comparable or even greater significance. This limitation can be mitigated through careful inspection of the intermolecular geometry in conjunction with analysis of IGMH isosurface volumes revealed using different isovalues. In particular, and in several cases, O···I close contacts are not identified by QTAIM, even though their presence is supported by geometrical parameters and IGMH analysis, indicating that they represent genuine, albeit weak, intermolecular interactions.

Our results also suggest that the aggregation process exhibits a clear transition from marginal stabilization at the dimer level to increasing destabilization in larger clusters, demonstrating negative cooperativity in which each added monomer progressively weakens the net stability of the assembly due to growing repulsive contributions.

The present results further suggest that the stability of the investigated anion–anion assemblies is sensitive to the dielectric environment. While highly polar media can effectively screen Coulombic repulsion and facilitate close anion–anion organization, less polar environments may alter the stability of the resulting dimers and oligomers, which may, in turn, influence their observed behavior and interpretation. Further investigation of solvent and dielectric effects on these assemblies would provide additional insight into the chemical physics and physical chemistry of anti-electrostatic interactions in condensed-phase systems.

The unusually large stabilization obtained for the (BrO_4_^−^)_2_ assembly within the SMD treatment further highlights the limitations of continuum dielectric models for highly charged anionic systems. In such cases, the absence of explicit local crystal-environment effects and the oversimplified treatment of dielectric screening may lead to artificial over-stabilization of weakly associated ion pairs. The result therefore should not be interpreted as evidence of intrinsically favorable anion–anion attraction, but rather as a manifestation of the sensitivity of these systems to the surrounding electrostatic environment and to the approximations inherent in implicit solvation models.

While this is the preliminary study clarifying the chemical contexts where a directionally oriented halogen-centered σ-hole driven close contact cannot be regarded as halogen bonds, we expect that the essence of this conclusion will be applicable to other elements of chalcogen, pnictogen, and tetrel family. 

## 6. Conclusions

This study examined the nature of anion–anion interactions in perhalate crystal structures using MESP, QTAIM, IGMH, NBO, and SAPT analyses. The results demonstrate that the XO_4_− dimers and oligomers observed in crystals are intrinsically unstable in the gas phase and become structurally viable only in dielectric environments that effectively screen Coulombic repulsion. Although directional X···O and O···O close contacts are observed, the halogen centers do not exhibit electrophilic σ-holes, even when higher isodensity surfaces are considered. Consequently, these interactions cannot be classified as halogen (or chalcogen) bonds within the accepted σ-hole framework, and their stabilizing or destabilizing character within an atom–atom diatomic framework remains to be fully elucidated. The observed assemblies are instead better described as environment-assisted anti-electrostatic associations governed by a balance of electrostatic screening, polarization, dispersion, and crystal packing effects. The results further indicate that the stability of these assemblies is sensitive to the surrounding dielectric environment. Further studies exploring solvent and medium effects on anti-electrostatic interactions in condensed-phase systems are underway and will be reported elsewhere.

## 7. Computational Methods

A number of crystal structures containing ClO_4_^−^ and IO_4_^−^ species as counterions were examined from the Cambridge Structural Database (CSD) [42,43]. The former typically exhibits zero- or one-dimensional arrangements, whereas the latter forms chain-like or higher-dimensional inorganic architectures. In contrast, only a limited number of crystal structures containing BrO_4_^−^ are currently known. Dimers and oligomers extracted from these crystals were subsequently energy-minimized using Gaussian 16 [44], followed by frequency calculations to confirm the nature of the stationary points. All calculations were performed using the M06-2X functional in conjunction with the def2-TZVPPD basis set obtained from the EMSL Basis Set Library [45,46].

Because these species are not stable in the gas phase due to strong electrostatic repulsion, the Solvation Model based on Density (SMD) [47] was employed throughout, with water as the solvent, using the Self-Consistent Reaction Field (SCRF) approach as implemented in Gaussian 16. The Polarizable Continuum Model (PCM) [48] was also applied in selected cases to assess its effect on the stability of the dimers.

To demonstrate the directional nature of negative σ-hole interactions, simple model systems such as CH_3_F, N_2_, and F_2_ were considered, and both linear and T-shaped dimers were examined in the gas phase, as well as in solution. In all cases, water was used as a solvent.

The Molecular Electrostatic Surface Potential (MESP) [27,28,49], the Quantum Theory of Atoms in Molecules (QTAIM) [30], and the Independent Gradient Model based on Hirshfeld partitioning (IGMH) [31,50] were employed. MESP analysis was used to characterize electrophilic and nucleophilic regions through the local most minimum and maximum of the electrostatic potential (*V_S,min_* and *V_S,max_*, respectively), and to assess whether the observed intermolecular interactions in the complexes can be classified as halogen bonds, chalcogen bonds, or instead represent anti-electrostatic interactions. QTAIM analysis was used to evaluate its effectiveness and limitations in identifying bonding interactions, and the results were compared with those inferred from intermolecular geometries and IGMH isosurface features. Both AIMAll [51] and Multiwfn [50] suite of codes were used.

Symmetry-Adapted Perturbation Theory (SAPT) [25,26] calculations were performed using the PSI4 program [52]; however, the energy decomposition is limited to the gas phase. Therefore, SAPT analysis at the SAPT2+3(CCD) level of theory was carried out on selected dimers using their energy-minimized SMD geometries to assess whether the interactions can be characterized as anti-electrostatic based on the interaction energies that were also compared with their corresponding binding energies (*E_b_*). The inclusion of the coupled-cluster doubles (CCD) treatment in SAPT2+(3)(CCD) is essential for an accurate description of dispersion interactions. CCD is a correlated wavefunction method that accounts for electron correlation through double excitations, providing a more reliable representation of long-range correlation effects compared to lower-level approaches such as MP2.

In addition, second-order perturbative charge-transfer analysis within the Natural Bond Orbital (NBO) framework [29,53,54] was performed in selected cases to elucidate the orbital contributions to the intermolecular interactions.

All periodic density functional theory calculations were performed using the projector augmented-wave (PAW) method [55] within the VASP code [56]. The exchange–correlation interactions were treated within the generalized gradient approximation (GGA), employing the PBE functional. A plane-wave cutoff of 520 eV was used to ensure convergence of total energies, forces, and stresses, and electronic self-consistency was achieved with a 10^−5^ eV energy criterion. Methfessel–Paxton smearing of order zero with a width of 0.05 eV was applied, suitable for insulating or wide-bandgap molecular crystals. Structural optimizations employed the conjugate-gradient algorithm until forces were below 0.03 eV Å^−1^. Brillouin zone sampling was performed using an appropriate 2 × 2 × 2 *k*-point mesh. Real-space projections were disabled to maintain full reciprocal-space precision. Long-range dispersion interactions were included via the DFT-D3 method with Becke–Johnson damping to capture van der Waals effects stabilizing the supramolecular structure.

## Figures and Tables

**Table 1:** SAPT component energies (kcal mol^−1^) for CH_3_F complexes of N_2_ and F_2_. Electrostatics, Exchange, Induction, and Dispersion are SAPT2-level components. Total SAPT2+3(CCD) = fully corrected interaction energy including higher-order and CCD corrections.

Complex	Geometry	Electrostatics	Exchange	Induction	Dispersion	Total SAPT2+3(CCD)
N_2_⋯FCH_3_	T-shaped	−0.33	0.37	−0.07	−0.52	−0.54
F_2_⋯FCH_3_	Linear	−0.01	0.37	−0.05	−0.53	−0.22
F_2_⋯FCH_3_	T-shaped	−0.39	0.36	−0.10	−0.52	−0.64

**Table 2:** SAPT-based dissected electrostatics, exchange, induction, and dispersion component energies, and total interaction energies of selected XO_4_^−^ dimers. Total SAPT2+3(CCD) refers to the fully corrected interaction energy including higher-order and CCD corrections. SMD-optimized geometries were used. All values are given in kcal mol^−1^.

Complex ^a,b^	Geometry	Electrostatics	Exchange	Induction	Dispersion	Total SAPT2+3(CCD)
(ClO_4_^−^)_2_ (ADAGPC)	Figure 3d	73.8	2.1	−4.0	−3.0	69.0
(BrO_4_^−^)_2_ (ADAWAG)	Figure 4b	53.7	0.0	−1.6	−0.5	51.7
(BrO_4_^−^)_2_ (ADAGPC)	Figure 4a	59.1	0.1	−2.1	−0.8	56.3
IO_4_^−^···IO_4_^−^(HOHMOG05)	Figure 6a	48.9	0.1	−1.8	−0.5	46.7
IO_4_^−^···IO_4_^−^ (JOJZAL)	Figure 6g	62.9	4.3	−4.3	−4.0	58.9
IO_4_^−^···IO_4_^−^ (BEKPAM)	Figure 6e	62.9	13.3	−5.7	−7.9	62.7

^a^ The names in parentheses correspond to crystal structures in the CSD from which the dimers were extracted and subsequently fully optimized using the SMD model. ^b^ The names in parentheses shown for the (BrO_4_^−^)_2_ dimers correspond to crystal structures containing ClO_4_^−^; in these cases, Cl was replaced by Br in the extracted dimers prior to optimization.

**Table 3:** Comparison of binding energy with the SAPT total energies (kcal mol^−1^) for CH_3_F complexes of CO, N_2_, HCN and H_2_CO. Electrostatics, Exchange, Induction, and Dispersion component energies are included. Total SAPT2+3(CCD) refers to the fully corrected interaction energy including higher-order and CCD corrections. The SMD geometries of the dimers were used.

Complex ^a^	Electrostatics	Exchange	Induction	Dispersion	Total SAPT2+3(CCD)	*E_b_* kcal mol^−1^)
H_3_CF⋯CO	0.44	0.34	−0.08	−0.43	0.27	−0.55
H_3_CF⋯OC	0.19	0.24	−0.06	−0.41	−0.04	−0.44
H_3_CF⋯N_2_	0.25	0.09	−0.03	−0.26	0.05	−0.23
H_3_CF⋯NCH	1.37	0.12	−0.09	−0.30	1.10	−0.21
H_2_CF⋯OCH_2_	1.41	0.22	−0.12	−0.43	1.08	−0.37

^a^ See Figure 9 for complexes.

## Data Availability

This research used data reported in the manuscript itself.
